# An unusual case of an extensive post-injection retroperitoneal abscess in an intravenous drug user

**DOI:** 10.1093/jscr/rjae398

**Published:** 2024-06-04

**Authors:** Georgios Gerasopoulos, Vasileios Patriarcheas, Angelos C Mitsas, Foteini Karagianni, Panagiotis Routis, Minas Kostis, Vasileios Voultsinos, Michail Lazaridis, Anastasios Tsitlakidis

**Affiliations:** General Surgery Department, Imathia General Hospital, Veria Unit, Papagou Settlement, Veria, Central Macedonia, Veria 59132, Greece; Internal Medicine Department, Imathia General Hospital, Veria Unit, Papagou Settlement, Veria, Central Macedonia, Veria 59132, Greece; General Surgery Department, Imathia General Hospital, Veria Unit, Papagou Settlement, Veria, Central Macedonia, Veria 59132, Greece; General Surgery Department, Imathia General Hospital, Veria Unit, Papagou Settlement, Veria, Central Macedonia, Veria 59132, Greece; General Surgery Department, Volos General Hospital, Volos 38222, Greece; Internal Medicine Department, Imathia General Hospital, Veria Unit, Papagou Settlement, Veria, Central Macedonia, Veria 59132, Greece; Diagnostic Radiology Department, Imathia General Hospital, Veria Unit, Papagou Settlement, Veria, Central Macedonia, Veria 59132, Greece; General Surgery Department, Imathia General Hospital, Veria Unit, Papagou Settlement, Veria, Central Macedonia, Veria 59132, Greece; General Surgery Department, Imathia General Hospital, Veria Unit, Papagou Settlement, Veria, Central Macedonia, Veria 59132, Greece

**Keywords:** retroperitoneal abscess, perirenal abscess, IV drug use

## Abstract

Retroperitoneal abscesses constitute an uncommon, complex, and life-threatening intra-abdominal infection. The insidious nature of the presentation, coupled with the presence of non-specific clinical symptoms, might result in misdiagnosis or delayed diagnosis, ultimately contributing to substantial morbidity and mortality. Herein we report a case of a 32-year-old intravenous drug user who presented to the emergency department complaining of high-grade fever, intense hiccough, and back pain due to retroperitoneal abscess formation after intravenous injection in the left femoral vein.

## Introduction

Retroperitoneal abscesses (RA) are rare and potentially fatal infections characterized by a gradual and subtle progression. Due to their insidious nature of presentation, which in turn leads to delayed diagnosis or misdiagnosis, they are associated with increased morbidity and mortality [1]. RA usually have an occult clinical presentation; signs and symptoms may include high-grade fever, fatigue, and abdominal and/or flank pain, whereas in some cases, clinical examination may reveal signs of subcutaneous emphysema [2, 3]. Of note, in some patients, extra-abdominal manifestations such as painful swellings in the groin and pain in the lower limb can be the only presenting symptoms. For this reason, the diagnosis of retroperitoneal abscesses requires a high degree of clinical suspicion. Among imaging modalities, computed tomography (CT) is the diagnostic method of choice. The treatment approach includes antibiotic therapy combined with drainage of the abscess to decrease mortality and morbidity, which can be achieved through either surgical intervention or percutaneous guided drainage. We present a case of an extended RA as a consequence of left femoral vein puncture in an intravenous drug user [2, 4].

## Case presentation

**Figure 1:**
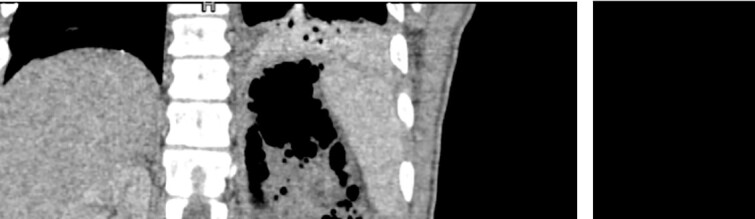
CT images of the abdomen (venous phase) revealed a protruberance in the left inguinofemoral region extending into the retroperitoneal space, progressing as a perirenal abcess, spreading to the left sub-diaphragmatic region and causing an air-fluid level and an infiltrate in the lower lobe of the lung.

A 32-year-old male with no known medical history presented to the emergency department complaining of a high-grade fever of up to 40°C with rigors, intense hiccough, and back pain started four days prior. The patient reported a long-standing intravenous drug use (IVDU). Upon arrival, he was febrile (T: 41°C) and tachypneic (24 breaths/minute), the following vital signs were: heart rate 134 bpm, blood pressure 116/73 mmHg, oxygen saturation 94%. Examination of the abdomen revealed tenderness in the left iliac fossa and an abscess was observed in the left inguinofemoral region. Laboratory investigations revealed a leukocytosis of 12.300/mm^3^, elevated inflammatory markers (C-reactive protein of 19.24 mg/dl), and procalcitonin of 39 ng/ml, whereas the rest of the laboratory parameters were within normal limits. The microscopic urine analysis was normal. Blood cultures were obtained from separate venipuncture sites, and subsequently, we began intravenous fluid administration. Among the possible diagnoses, retroperitoneal abscess was prioritized due to clinical findings as well as because the patient reported several femoral vein punctures for drug injection. To set the diagnosis, the patient underwent a CT scan with IV and oral contrast. CT scan revealed swelling of the left femoral groin with the presence of a retroperitoneal abscess expanding through the left iliopsoas muscle to the perirenal space and left hemidiaphragm, which resulted in small left pleural effusion and infiltrates in the left lower lobe (Fig. 1).

**Figure 2:**
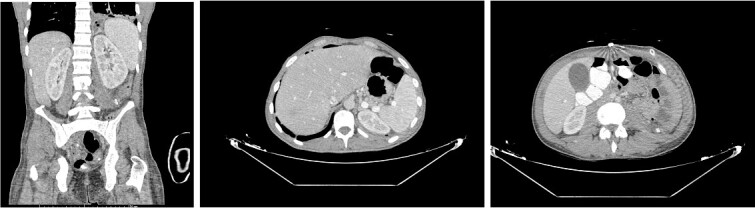
Postoperative CT done on the fourth day after surgery revealed a significant reduction in abcess size and improvement of the left lower lung lobe infiltration.

The patient was then admitted to the General Surgery department and treatment with broad-spectrum antibiotics (piperacillin/tazobactam, vancomycin, and metronidazole) was initiated. On the second day of hospitalization, the patient was led to the operating room, where the retroperitoneal cavity was exposed through a midline incision and a large amount of pus was drained. The retroperitoneal and peritoneal cavities were thoroughly irrigated, and three vacuum drains were placed. A pus culture was also obtained. On the fourth postoperative day, a new CT scan (Fig. 2) was performed, which revealed a significant decrease of the abscess as well as a significant reduction of the free fluids in the retroperitoneal area. Our patient showed a gradual clinical and laboratory improvement, and he was discharged on the eighth postoperative day. At the follow-up (3 months later), the patient’s overall health was satisfactory with no signs of a recurrence of the retroperitoneal abscess.

## Discussion

The retroperitoneal space is described as a potential space between the peritoneum and the transversalis fascia, lining the posterior abdominal cavity, which extends from the diaphragm superiorly to the pelvic brim inferiorly and the margin of the quadratus lumborum muscles laterally [5, 6]. RA can be classified as either primary or secondary based on their origin [7]. Secondary abscesses are more common and arise from an infection or perforation in a nearby organ of the urinary or gastrointestinal system, whereas primary are the result of an infection spreading through the bloodstream (Table 1) [1, 7]. Regarding the microbiology, retroperitoneal abscesses can be monomicrobial or polymicrobial. Common causal pathogens include *Escherichia coli, Staphylococcus aureus, Pseudomonas aeruginosa, Clostridium perfigens*, and other anaerobes [1, 8]. In our patient, *P. aeruginosa* was the isolated pathogen from cultures obtained during the surgical operation.

**Table 1:** Most common causes of secondary RA [10, 11].

Infections	Urinary tract system infections, vertebral osteomyelitis, necrotizing soft tissue infection acute colonic diverticulitis, acute pancreatitis, retrocecal appendicitis
Perforation	Ascending and descending colon, rectum (malignancies, acute colonic diverticulitis), Perforation of the duodenum (peptic ulcer disease), inflammatory bowel disease, surgery, or trauma

Of note, our patient did not report abdominal pain, and upon his arrival, the main complaining symptoms were fever, back pain, and hiccough (due to hemidiaphragm irritation), highlighting the subtle and non-specific clinical presentation of retroperitoneal abscesses, which can impede timely diagnosis and precise treatment [9].

CT is considered the most reliable and accurate method for the diagnosis of RA, offering precise localization of the infection and revealing the correlation between lesions and adjacent organs [1, 3]. The treatment approach for retroperitoneal abscesses consists of source control of infection, the use of wide-spectrum antibiotics, and nutritional support. The most critical aspect is the timely and effective control of the source of infection, with the goal of eliminating the infected suppurative and necrotic tissue [1]. This can be achieved either by percutaneous puncture drainage guided by B-mode ultrasound/CT or by surgical drainage. Despite the fact that percutaneous drainage is the preferred approach for the majority of patients, in cases where the spread of infection is too extensive (abscess size of >5 cm) or percutaneous drainage can not adequately control the infection, invasive surgery should be considered [4, 12, 13]. In our case, due to the size of the abscess (>5 cm), we performed open surgical drainage through a midline incision and placed vacuum drains which were removed on the fifth postoperative day.

In our case, the patient had a history of longstanding IVDU, and he also reported that he performed several femoral vein punctures for drug injection, which leads to the conclusion that the retroperitoneal abscess was of primary origin.

## Conclusion

RA exhibit an insidious onset with subtle signs and symptoms. Early and accurate diagnosis is crucial, and clinicians should have a high degree of suspicion of this entity, which should always be included in the differential diagnosis of fever in IVDU patients. A better understanding of this rare clinical entity ought to increase consciousness, resulting in an early treatment approach, which consequently would improve clinical outcomes.
